# Effects of tobacco exposure on perinatal suicidal ideation, depression, and anxiety

**DOI:** 10.1186/s12889-016-3254-z

**Published:** 2016-07-22

**Authors:** Shu-Chuan Weng, Jian-Pei Huang, Ya-Li Huang, Tony Szu-Hsien Lee, Yi-Hua Chen

**Affiliations:** School of Public Health, College of Public Health, Taipei Medical University, Taipei, Taiwan; Department of Obstetrics and Gynecology, Mackay Memorial Hospital, Taipei, Taiwan; Mackay Memorial Hospital, New Taipei, Taiwan; MacKay Junior College of Medicine, Nursing, and Management, Taipei, Taiwan; Department of Public Health, School of Medicine, College of Medicine, Taipei Medical University, Taipei, Taiwan; Department of Health Promotion and Education, National Taiwan Normal University, Taipei, Taiwan

**Keywords:** Smoking, Secondhand smoke exposure, Pregnancy, Suicidal ideation, Depression

## Abstract

**Background:**

Previous studies have stressed the importance of tobacco exposure for the mood disorders of depression and anxiety. Although a few studies have focused on perinatal women, none have specifically considered the effects of smoking and secondhand smoke exposure on perinatal suicidal ideation. Thus, this study aimed to investigate the relationships of smoking/secondhand smoke exposure status with suicidal ideation, depression, and anxiety from the first trimester to the first month post partum.

**Methods:**

This cross-sectional study based on self-reported data was conducted at five hospitals in Taipei, Taiwan from July 2011 to June 2014. The questionnaire inquired about women’s pregnancy history, sociodemographic information, and pre-pregnancy smoking and secondhand smoke exposure status, and assessed their suicidal ideation, depression, and anxiety symptoms. Logistic regression models were used for analysis.

**Results:**

In the 3867 women in the study, secondhand smoke exposure was positively associated with perinatal depression and suicidal ideation. Compared with women without perinatal secondhand smoke exposure, women exposed to secondhand smoke independently exhibited higher risks for suicidal ideation during the second trimester (odds ratio (OR) = 7.63; 95 % confidence interval (CI) = 3.25–17.93) and third trimester (OR = 4.03; 95 % CI = 1.76–9.23). Women exposed to secondhand smoke had an increased risk of depression, especially those aged 26–35 years (OR = 1.71; 95 % CI = 1.27–2.29).

**Conclusions:**

Secondhand smoke exposure also considerably contributes to adverse mental health for women in perinatal periods, especially for the severe outcome of suicidal ideation. Our results strongly support the importance of propagating smoke-free environments to protect the health of perinatal women.

**Electronic supplementary material:**

The online version of this article (doi:10.1186/s12889-016-3254-z) contains supplementary material, which is available to authorized users.

## Background

Tobacco smoking is a major concern because of its harm to human health, especially that of perinatal women. Both active and passive smoking during pregnancy have been associated with negative impacts on maternal and infant health, including stillbirths, preterm deliveries, low birth weight, and neonatal death [1–5]. However, even during pregnancy, the husbands of more than half of women have a smoking habit [6]. Furthermore, 6.2 and 54.6 % of people are frequently exposed to secondhand smoke in indoor and outdoor public areas, respectively [7]. This means that women have a high chance of being exposed to a secondhand smoke environment.

Emotional disturbance is a crucial health consideration, especially during the perinatal period. The development of depression or anxiety is often preceded by specific or chronic life stressors (eg, pregnancy and motherhood in the case of perinatal women). Empirical studies have suggested that between 15 and 25 % of pregnant women experience anxiety or depression [8]. Indeed, women experience substantial hormonal and physiological changes during pregnancy. It was reported that the functioning of the hypothalamic–pituitary–adrenal (HPA) axis, which is key to stress response, changes dramatically during pregnancy, largely because of the influence of the placenta [9]. As pregnancy progresses, placental production of corticotropin-releasing hormone (CRH) increases exponentially [10]. CRH has been proposed as being involved in the pathophysiological response of the HPA axis in mental pathologies such as depression [11, 12].

Both depression and anxiety have been found to be significantly related to smoking [13–15]. Prospective cohort and case–control studies have also reported significant evidence that smoking is associated with all forms of suicidality [16–18]. Studies have also determined that secondhand smoke exposure is positively associated with depressive symptoms [19, 20]. However, the relationship between anxiety and secondhand smoke exposure was less consistent [21]. Indeed, either through active or passive smoking, exposure to various psychoactive compounds of tobacco smoke may contribute to the dysregulation of affective states [22]. For example, nicotine may affect numerous neurotransmitters to influence the pathophysiology of depression [23] through the activation or desensitization of nicotinic acetylcholine receptors (nAChRs) [24, 25]. Along with facilitating cholinergic neurotransmission, nAChRs affect the activities of the neuroendocrine system and are thus involved in depression and the HPA axis [26]. Specifically for nonsmokers, secondhand smoke exposure may lead to lower levels of dopamine and γ-aminobutyric acid (GABA), which have been related to an increased risk for mental disorder [27].

Most studies of perinatal women that have discussed smoking and secondhand smoke exposure have focused on the physical damage to the women and their children [28–31]. Several studies have found that pregnant women who were smokers or had quit after pregnancy were more likely to report depressive symptoms and anxiety disorders [32–35]. Few studies have investigated the link between smoking and suicidal behavior for postnatal women. Among those that have, Tavares et al. in 2012 found nonsignificant differences between smoking and suicidal behavior after adjusting for sociodemographics (eg, age and education), psychosocial factors (eg, social support and stressful events), and comorbidities of depression and anxiety disorder [36]. No study has specifically investigated the association between secondhand smoke exposure and suicidality among perinatal women.

A large body of literature relates to perinatal psychopathology, including depressive symptoms [37, 38], anxiety [39, 40], and suicidality [41, 42], with the effects of a history of depression before pregnancy being a frequent topic [43]. However, to the best of our knowledge, no researchers have simultaneously considered the effects of smoking and secondhand smoke exposure on perinatal suicidal ideation, depression, and anxiety. Furthermore, the associations have not been carefully examined during the pregnancy-to-postpartum periods.

The perinatal period encompasses the first trimester to the postpartum phase, with each constituent period marked by specific maternal mental and physical changes, fetal developments, and subsequent various clinical and practical implications regarding antenatal care and interventions [44]. Indeed, women in each trimester of pregnancy would experience various physical and mental symptoms. For example, at the earlier stage, mothers with syndromes of nausea and vomiting, the most common physical discomforts during the first trimester [45], may be more sensitive to tobacco exposure. Mental ailments are likely to increase as the pregnancy moves towards the third trimester [46]. During postnatal periods, mothers may display more emotional disturbances under tobacco exposure due to concerns of its impact on their infant’s health. Investigating variation of emotional symptoms at different periods of time may thus facilitate the design and implementation of intervention and health education programs at the most suitable times.

Thus, in this study, we investigated the relationships of smoking/secondhand smoke exposure status with suicidal ideation, depression, and anxiety from the first trimester to the first month post partum. Specifically, this study aimed to (1) examine the occurrence of suicidal ideation, depression, and anxiety in pregnant women from the prenatal to postpartum periods; (2) investigate the risks of suicidal ideation, depression, and anxiety among perinatal women who are exposed to tobacco, including pre-pregnancy smoking and secondhand smoke; and (3) explore whether associations between women’s exposure to tobacco and risks for suicidal ideation, depression, and anxiety differed according to the women’s characteristics.

## Methods

### Participants and procedures

This cross-sectional study was conducted in five hospitals in Taipei City and New Taipei City, Taiwan. The hospitals were a part of major obstetric hospitals in Northern Taiwan and were selected according to their willingness to participate in this perinatal mental health program. Between July 2011 and June 2014, pregnant women and women within 1 month post partum who had received outpatient service at one of the hospitals were consecutively approached and invited to participate in the study. The exclusion criteria were (1) being unable to read or write Chinese and (2) having a severe psychiatric illness (ie, being clinically diagnosed with bipolar disorder, nonaffective psychosis (such as schizophrenia), or substance use disorder).

Interviewers were trained for standardization. The interviewers approached potential participants and explained to them the study and the content of the consent form. After providing informed consent, the participants spent approximately 15 min completing the questionnaire at the outpatient center, with an overall response rate of 75 %. The self-reported questionnaire inquired about the participants’ pregnancy history, sociodemographic information, and smoke and secondhand smoke exposure status, and assessed their suicidal ideation, depression, and anxiety symptoms.

### Measures

#### Smoking and secondhand smoke status

We attempted to collect information on tobacco smoking during pregnancy. However, the number of reported prenatal smokers was too low for further analysis because smoking during pregnancy is a violation of Taiwanese law. Accordingly, we only used pre-pregnancy smoking status (which women were more comfortable to report accurately) to estimate the association between smoking behavior and mood disorders. Pre-pregnancy smoking status was assessed using the following questions: “Have you smoked more than 100 cigarettes over your entire life?” If the answer was “yes,” then the participant was asked, “How many cigarettes per day did you smoke immediately before your pregnancy?” If the answer was more than one cigarette per day, then the participant was identified as a pre-pregnancy smoker [47].

Secondhand smoke exposure status was assessed using the question: “Does anyone smoke around you in your home or workplace?” If the answer was “always” or “usually,” then the participant was identified as having “high secondhand smoke exposure.” If the answer was “sometimes” or “never,” then the participant was identified as having “low secondhand smoke exposure.”

#### Depression and anxiety

The Edinburgh Postnatal Depression Scale (EPDS) was used to assess symptoms of depression in the participants. The EPDS has been reported to efficiently screen for depression in women during the pregnancy and postnatal periods [48, 49]. The EPDS consists of ten items that assess how participants have been feeling in the past 7 days. The total EPDS score is calculated by summing participant responses; scores range from 0 to 30, with higher scores reflecting a greater level of depressive symptoms. In this study, EPDS scores of >13 were identified as indicating depression [48, 50]. The Chinese version of the EPDS has exhibited strong reliability and validity [51, 52].

Anxiety was assessed using the State-Trait Anxiety Inventory (STAI) [53]. The STAI is a 20-item questionnaire with total scores ranging from 20 to 80, with higher scores indicating a higher level of anxiety. In this study, a total STAI score of >60 was identified as indicating anxiety [54]. The Chinese version of the STAI exhibited strong reliability and validity [55].

#### Suicidal ideation

Similar to previous studies, suicidal ideation was defined as an answer of “sometimes” or “yes, quite often” to question 10 of the EPDS: “The thought of harming myself has occurred to me.” “No suicidal ideation” was defined by answering “hardly ever” or “never” to question 10 [56].

#### Other covariates

Other covariates that were reported as being associated with smoking and mood disorders were recruited for further consideration, namely peripartum period (first trimester, second trimester, third trimester, and first month post partum), age group (<25, 26–35, and >36 years), marital status (married vs. other), monthly income (<NT$30,000, NT$30,000–100,000, and > NT$100,000), employment status (yes vs. no), educational level (<12, 12–15, >15 years of education), body mass index (BMI) [57], birth order (primipara vs. multipara), baby sex (male, female, or unknown), agreement with the expectation of fetus sex (inconsistent vs. other (including consistency, no specific expectation, and unknown sex) [58], planned pregnancy (yes vs. no), and a history of depression prior to pregnancy (yes vs. no) [35]. The participants who responded to the question “How was your quality of sleep during the past 7 days?” with “very good,” “good,” or “normal” were identified as not having sleep problems. Those who answered “bad” or “very bad” were identified as having sleep problems.

### Statistical analysis

All available demographic measures were considered in the analysis. The occurrence of suicidal ideation, depression, and anxiety were calculated separately for the four perinatal periods including the first to third trimesters during pregnancy and 1 month post partum. Associations of smoking and secondhand smoke exposure status with each dependent variable of suicidal ideation, depression, and anxiety were assessed through chi-squared tests.

A univariate logistic regression was performed as the first step to estimate the risks of suicidal ideation, depression, and anxiety associated with smoking and secondhand smoke exposure status. Variables which may possibly be associated with dependent variables (*p* < 0.1) were further considered in multivariate logistic regression models, with odds ratios (ORs) and 95 % confidence intervals (CIs) estimated. Factors such as BMI, birth order, the baby’s sex, and agreement with the expectation of the baby’s sex that did not display significant effects were not included in the final model for estimation. In addition, the potential modifying effects of demographic factors were considered. Subgroup analyses and models were applied if the interaction terms of the secondhand smoking status and demographic factors were associated with the dependent variables (*p* < 0.2). In the suicidal ideation model, a significant interaction existed between secondhand smoke exposure status and perinatal period. In the depression model, a significant interaction existed between secondhand smoke exposure status and age group. No other variables showed significant modifying effects.

The significance level for all statistical analyses was *p* < 0.05. All data were analyzed using statistical software of SAS version 9.3 (SAS, Cary, NC, USA).

## Results

In total, 3867 participants completed the questionnaire. Table 1 presents the sample characteristics. Approximately three-fourths of the women were in the second (34.83 %) and third trimesters (40.35 %). Most of the participants were 26–35 years old (73.93 %), employed (76.92 %), married (97.07 %), had a monthly income of NT$30,000–NT$100,000 (63.61 %), and had been educated for >12 years (86.03 %). Approximately 60 % of the sample were primipara.

**Table 1:** Characteristics of studied perinatal women in Taiwan (*n* = 3867)

Variable		Number^b^	Percent
Pregnancy	1st trimester	710	18.40
	2nd trimester	1344	34.83
	3rd trimester	1557	40.35
	Postpartum 1 m	248	6.43
Age (years)	≤25	173	4.47
	26 ~ 35	2859	73.93
	≥36	835	21.59
Employment status	No	886	23.08
	Yes	2952	76.92
Marital status	Married	3747	97.07
	Single and others	113	2.93
Monthly income (NT$)^a^	<30,000	151	3.94
	30,000 ~ 100,000	2437	63.61
	>100,000	1243	32.45
Educational level	<9 years	886	1.19
	9 ~ 12 years	2952	12.78
	>12 years	886	86.03
Parity	1	2194	59.51
	2+	1493	40.49

^a^The exchange rate on June 30, 2013 was US$1.00 = NT$30.19 (New Taiwan dollars)

^b^Total counts may vary because of missing data

**Fig. 1:**
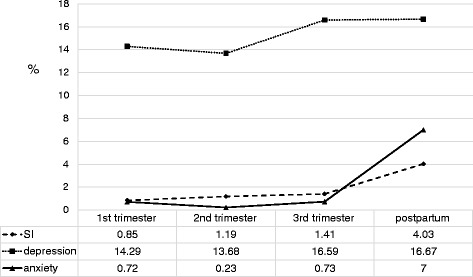
Reports of suicidal ideation, depression, and anxiety occurrences among women from the first trimester to 1 month postpartum

Figure 1 presents the suicidal ideation, depression, and anxiety during the four perinatal periods. More women reported depression than other outcomes of anxiety and suicidal ideation. Suicidal ideation increased with time (0.85–4.03 %) and was higher in the postpartum period. The highest occurrences observed for suicidal ideation, depression, and anxiety were during 1 month post partum.

Table 2 shows that the women who had high secondhand smoke exposure were more likely to have suicidal ideation and depression (*p* < 0.001) but not anxiety. Regarding pre-pregnancy smoking status, the women who were pre-pregnancy smokers were more likely to have suicidal ideation (*p* = 0.0032) but not depression or anxiety.

**Table 2:** Effects of tobacco exposure on suicidal ideation, depression, and anxiety among perinatal women

	Total	Suicidal ideation		Non-suicidal ideation		*p* value	Depression		Non-depression		*p* value	Anxiety		Non-anxiety		*p* value
		*n*	%	*n*	%		*n*	%	*n*	%		*n*	%	*n*	%	
Secondhand smoke exposure status																
High exposure	505	18	3.57	486	96.43	<0.0001	120	23.95	381	76.05	<0.0001	4	0.82	486	99.18	1
Low exposure	3187	35	1.1	3141	98.9		430	13.6	2731	86.4		31	1	3065	99	
Smoking status immediately before pregnancy																
Smokers	169	7	4.14	162	95.86	0.0032	32	18.93	137	81.07	0.1469	0	0	166	100	0.626
Non-smokers	3475	36	1.04	3427	98.96		512	14.85	2936	85.15		24	0.71	3347	99.29	

### Suicidal ideation

The effect of secondhand smoke exposure on suicidal ideation was statistically significant. After adjustment for perinatal period, age, marital status, monthly income, employment status, educational level, planned pregnancy, history of depression, and sleep quality, the OR for suicidal ideation for the women who had high secondhand smoke exposure was 2.5 (95 % CI = 1.30–4.82) (see Additional file 1). In the multivariate analysis, we found that women who were younger, had a history of depression, or were not sleeping well were significantly more likely to have suicidal ideation.

To further explore potential effects by different perinatal periods, a subgroup analysis and models were performed for each time period (Table 3). The effect of secondhand smoke exposure on suicidal ideation was significant in the second (OR = 7.63; 95 % CI = 3.25–17.93) and third trimesters (OR = 4.03; 95 % CI = 1.76–9.23). This means that pregnant women during the second or third trimesters who were frequently exposed to secondhand smoke had an increased risk of suicidal ideation. The model in the postpartum period was unavailable because no participant with high secondhand smoke exposure reported suicidal ideation.

**Table 3:** Effects of perinatal secondhand smoke exposure on suicidal ideation by perinatal period^b^ and on perinatal depression by age according to logistic regression models

Suicidal ideation vs. Perinatal period^a^	1^st^ trimester		2^nd^ trimester		3^rd^ trimester	
	OR	95 % CI	OR	95 % CI	OR	95 % CI
High secondhand smoke exposure	1.39	0.16 ~ 11.91	7.63**	3.25 ~ 17.93	4.03*	1.76 ~ 9.23
Depression vs. Age (years)^c^	≤25		26 ~ 35		≥36	
	OR	95 % CI	OR	95 % CI	OR	95 % CI
High secondhand smoke exposure	2.05	0.80 ~ 5.22	1.71***	1.27 ~ 2.29	0.84	0.42 ~ 1.67

*p < 0.05; **p < 0.01; ***p < 0.001. OR, odds ratio; CI confidence interval

^a^Model was adjusted for perinatal period, marital status, monthly income, employment status, educational level, planned pregnancy, history of depression, sleep problems, and secondhand smoking

^b^The postpartum data were invalid because no participant exposed to secondhand smoke reported suicidal ideation

^c^Model was adjusted for perinatal period, marital status, monthly income, employment status, educational level, planned pregnancy, history of depression, and sleeping problems

### Depression

Exposure to secondhand smoke was also associated with depression. After adjustment for other covariates, the OR for depression among the women who had high secondhand smoke exposure was 1.55 (95 % CI = 1.20–2.01) (see Additional file 1). In the multivariate analysis, we determined that women who were unmarried, had a lower income, had an unplanned pregnancy, had a history of depression, or were not sleeping well were significantly more likely to have depression.

The interaction effect of age and secondhand smoking was significant (*p* = 0.02). To further explore the potential modifying effects in different age groups, a subgroup analysis and models were applied for each age group (Table 3). The effect of secondhand smoke was only significantly associated with depression in women aged 26–35 years (OR = 1.71; 95 % CI = 1.27–2.29).

### Anxiety

We found no significant association between secondhand smoke and anxiety (see Additional file 1). However, in the multivariate analysis, we observed that postpartum women and women who had a lower education level were significantly more likely to report higher levels of anxiety.

## Discussion

The current study determined that secondhand smoke exposure was positively associated with perinatal depression and suicidal ideation. Compared with women who had low secondhand smoke exposure, those exposed to secondhand smoke more frequently during the second and third trimesters exhibited a 4–7-fold higher risk of suicidal ideation, and women exposed to secondhand smoke more frequently and who were aged younger than 26–35 years had an increased risk of depressive symptoms.

We also found that secondhand smoke exposure was associated with depression, which is consistent with the findings of previous studies [59, 60]. The impact of secondhand smoke exposure on depressive symptoms especially affected women aged 26–35 years. No significance was observed for women aged 35 years or more. Previous studies have shown that younger antenatal and postnatal women have higher risks of experiencing depressive symptoms [61–63]; older women are believed to have the ability to adapt to changes in the external environment and to have fewer mood disorders [64]. The nonsignificant association between secondhand smoke exposure and depression for women aged <25 years in our study is probably due to the small sample size and its limited statistical power. More studies are required to clarify this relationship.

This is the first study to observe that women with secondhand smoke exposure more frequently had an increased risk of perinatal suicidal ideation, especially during the second and third trimesters. Two reasons could explain the difference among trimesters. First, approximately 87 % of pregnant women complain of nausea and vomiting during the first trimester [45]. Women may have an aversion to being exposed to secondhand smoke early in pregnancy because of physiological changes. Thus, they may attempt to limit their exposure to secondhand smoke. In addition, Peacock et al. in 1998 found a strong positive correlation between cotinine measures at 14 and 28 weeks [65]. Chiu [66] determined that urinary and serum cotinine levels were elevated as gestation progressed. It seems that pregnant women may experience different effects of secondhand smoke exposure throughout the 3 trimesters.

Certain mechanisms may explain the association between secondhand smoke exposure and depressed mood. Tobacco smoke affecting the neurotransmitter systems of pregnant passive smokers may reflect the role of neurotransmitter pathways in the biological mechanism of depression. Nicotine intensity may increase the plasma levels of CRH and adrenocorticotropic hormone (ACTH), resulting in the secretion of more cortisol, which consequently affects mood, cognition, and behavior by changing the availability of brain neurotransmitters [67]. Studies have further suggested that secondhand smoke exposure may reduce the levels of dopamine and GABA. These two biochemicals have been linked to depression [20, 34]. Additionally, passive smoking may also provoke a feeling of withdrawal, a symptom associated with depressed mood and major depressive episodes through the mechanism of augmented monoamine oxidase A binding in the brain [68].

Moreover, we found that pre-pregnancy smoking status was associated with suicidal ideation. In some prospective studies, smoking could explain suicidality risk more accurately than could other variables [69, 70]. Our study further identified that pre-pregnancy smoking status was a crucial risk factor for suicidal ideation among perinatal women. Previous studies have shown that smoking and suicidal behaviors are positively associated with sensation seeking [71, 72]. As both smoking and suicidal behaviors could be considered risk-taking behaviors, the link between suicidal ideation and smoking warrants more investigation in future studies.

Our results presented a null relationship between pre-pregnancy smoking and perinatal depression and anxiety. Although some studies have observed an association between smoking and depression [59, 73, 74], the link between smoking and anxiety was less clear [21]. Consistent with our null findings, a cross-sectional study in the Netherlands determined that anxiety and depressive symptoms were not associated with continued smoking for pregnant women [75]. Examining smoking intake might aid in clarifying the relationship between smoking and mood disorders; however, the number of active smokers during pregnancy in our study was too small for such analysis. In addition, smoking among women, especially during the perinatal period, is a sensitive topic. Women might feel hesitant to report their smoking behaviors, which could bias results toward the null. Smoking may jointly act with other physical and psychosocial factors to affect perinatal mood disorders. Future studies are suggested to recruit more active smokers during the perinatal period and to classify them by smoking intake for further examination.

There is a general awareness that tobacco exposure endanger maternal and fetal health. In our investigation, we found that pre-pregnancy smoking and secondhand smoke exposure increased risks to a woman's mental health during the perinatal period. Our investigation provides a compelling reason that we should advocate more restricted smoke-free legislation to prevent perinatal women from being exposed to secondhand smoke at home and in the workplace, especially during the second and third trimesters, to avoid adverse mental health outcomes. Appropriate intervention strategies to create smoke-free environments in the home and public places for perinatal women are essential. Huang et al. [76] used a transtheoretical model for preventive behavior against passive smoking by perinatal women. We suggest that this idea is expanded on by future studies and strategies.

This study had several strengths. First, to our knowledge, this was the first study to focus on the effects of secondhand smoke exposure on perinatal suicidal ideation. Second, this study used a larger sample than did previous studies on the effects of smoke and mental status during the perinatal period [32, 74, 75]. Third, the current study collected data from the entire perinatal period from the first trimester to 1 month post partum. Finally, we assessed the effects of pre-pregnancy smoking and secondhand smoke exposure on perinatal mental status simultaneously and adjusted for other risk factors that might have interfered with the mental status of the participating perinatal women.

The current study also had some limitations. First, this study used self-reported tobacco exposure and lacked biochemical assay data. This might have caused a bias because of differences in individual subjective recognition and recall. However, according to Chiu et al. [77], cotinine levels in the urine and blood of pregnant women were significantly correlated with their self-reported information provided using a questionnaire. Thus, the self-reported information in the current study can be considered to be fairly accurate. Nevertheless, because the Taiwan law prohibits maternal smoking during pregnancy and because of the stigma associated with female smoking in Taiwan, the accuracy of the assessment of smoking during or immediately before pregnancy should be considered carefully. Second, this study used a convenience sample from five cooperating hospitals, which might limit the generalizability of our results to all perinatal women in Taiwan. Third, some risk factors that may have influenced the perinatal mental condition were not included in our model, such as stressful events and physical illnesses. This may have biased our results. Finally, the wide 95 % CIs specifically for the effects of secondhand smoke exposure status on suicidal ideation were probably due to the small sample size of women with suicidal ideation and exposure of secondhand smoking during the perinatal period. Thus, the statistical power of these results might be limited.

## Conclusion

Prenatal and postnatal women exposed to passive smoking are at risks of suicidal ideation and depressive symptoms, especially during the second and third trimesters and among younger women. This study extended knowledge of the effects of secondhand smoke exposure on perinatal mental health. In addition, our results support the importance of creating smoke-free environments for perinatal women. Further studies are suggested to elucidate the underlying mechanisms of the association between secondhand smoke exposure and mental health among different trimesters and ages.

## Abbreviations

ACTH, adrenocorticotropic hormone; BMI, body mass index; CI, confidence interval; CRH, corticotropin-releasing hormone; EPDS, Edinburgh Postnatal Depression Scale; GABA, γ-aminobutyric acid; OR, odds ratio; STAI, State-Trait Anxiety Inventory

## Additional file

Additional file 1: Multivariate analysis of risk factors for suicidal ideation, depression, and anxiety among perinatal women in Taiwan. (DOCX 20 kb)
