# Supplementary material for: Effects of tobacco exposure on perinatal suicidal ideation, depression, and anxiety
Source: BMC Public Health. 2016 Jul 22;16:623. doi: 10.1186/s12889-016-3254-z (PMC4957348; doi:10.1186/s12889-016-3254-z)
Supplement: Additional file 1: — Multivariate analysis of risk factors for suicidal ideation, depression, and anxiety among perinatal women in Taiwan. (DOCX 20 kb) [file 12889_2016_3254_MOESM1_ESM.docx]

**Additional file 1 Multivariate analysis of risk factors for suicidal ideation, depression, and anxiety among perinatal women in Taiwan**

| **Variable *N*=3890** | | **Suicidal ideation** | | | | **Depression** | | | | **Anxiety ^a^** | | | |
| --- | --- | --- | --- | --- | --- | --- | --- | --- | --- | --- | --- | --- | --- |
|  |  | Adjusted  OR | 95% CI | | | Adjusted  OR | 95% CI | | | Adjusted  OR | 95% CI | | |
| **Secondhand  smoke exposure**  **status** | Low exposure (ref.) |  |  |  |  |  |  |  |  |  |  |  |  |
|  | High exposure | 2.50** | 1.30 | ~ | 4.82 | 1.55*** | 1.20 | ~ | 2.01 | 0.882 | 0.248 | ~ | 3.138 |
| **Period** | 1st trimester (ref.) |  |  |  |  |  |  |  |  |  |  |  |  |
|  | 2nd trimester | 1.09 | 0.41 | ~ | 2.93 | 0.89 | 0.67 | ~ | 1.18 | 0.295 | 0.07 | ~ | 1.252 |
|  | 3rd trimester | 1.25 | 0.48 | ~ | 3.24 | 1.02 | 0.77 | ~ | 1.34 | 0.793 | 0.262 | ~ | 2.395 |
|  | postpartum | 3.38 | 0.74 | ~ | 15.43 | 1.34 | 0.68 | ~ | 2.64 | 11.282*** | 2.945 | ~ | 43.224 |
| **Age (years)** | ≤25 | 2.91* | 1.21 | ~ | 6.97 | 1.11 | 0.71 | ~ | 1.73 | 1.302 | 0.235 | ~ | 7.217 |
|  | 26~35 (ref.) |  |  |  |  |  |  |  |  |  |  |  |  |
|  | ≥36 | 0.80 | 0.34 | ~ | 1.89 | 0.94 | 0.73 | ~ | 1.22 | 0.859 | 0.284 | ~ | 2.595 |
| **Marital status** | Married |  |  |  |  |  |  |  |  |  |  |  |  |
|  | Other | 2.03 | 0.69 | ~ | 6.15 | 1.71* | 1.05 | ~ | 2.79 | - | - |  | - |
| **Monthly income (NT$) ^b^** | <30,000 (ref.) |  |  |  |  |  |  |  |  |  |  |  |  |
|  | 30,000~100,000 | 1.22 | 0.42 | ~ | 3.49 | 0.54** | 0.36 | ~ | 0.83 | 2.358 | 0.214 | ~ | 25.98 |
|  | >100,000 | 0.54 | 0.15 | ~ | 1.97 | 0.36*** | 0.23 | ~ | 0.57 | 2.398 | 0.195 | ~ | 29.506 |
| **Employment status** | No (ref.) |  |  |  |  |  |  |  |  |  |  |  |  |
|  | Yes | 0.56 | 0.300 | ~ | 1.06 | 0.86 | 0.69 | ~ | 1.08 | 0.864 | 0.333 | ~ | 2.237 |
| **Educational level** | <9 years (ref.) |  |  |  |  |  |  |  |  |  |  |  |  |
|  | 9~12 years | 0.81 | 0.16 | ~ | 4.01 | 0.61 | 0.28 | ~ | 1.36 | 0.172 | 0.028 | ~ | 1.065 |
|  | >12 years | 0.42 | 0.09 | ~ | 2.08 | 0.65 | 0.30 | ~ | 1.42 | 0.079** | 0.014 | ~ | 0.455 |
| **Planned pregnancy** | Yes (ref.) |  |  |  |  |  |  |  |  |  |  |  |  |
|  | No | 1.59 | 0.84 | ~ | 3.00 | 1.79*** | 1.47 | ~ | 2.20 | 0.652 | 0.251 | ~ | 1.69 |
| **Depression history** | No (ref.) |  |  |  |  |  |  |  |  |  |  |  |  |
|  | Yes | 3.85** | 1.54 | ~ | 10.89 | 2.12** | 1.33 | ~ | 3.37 | - | - |  | - |
| **Sleep problems** | No (ref.) |  |  |  |  |  |  |  |  |  |  |  |  |
|  | Yes | 2.80** | 1.48 | ~ | 5.300 | 2.54*** | 2.08 | ~ | 3.10 | 0.991 | 0.411 | ~ | 2.388 |

* *p* < 0.05; ** *p* < 0.01; *** *p* < 0.001. OR, odds ratio; CI, confidence interval; ref., reference.

a. For the anxiety model, because the marital status and history of depression cells were empty, these 2 variables were not included in the model.

b. The exchange rate on June 30, 2013 was US$1.00 = NT$30.19 (New Taiwan dollars).
